# Artificial Intelligence for Pigment Classification Task in the Short-Wave Infrared Range

**DOI:** 10.3390/s21186150

**Published:** 2021-09-13

**Authors:** Emeline Pouyet, Tsveta Miteva, Neda Rohani, Laurence de Viguerie

**Affiliations:** 1 Laboratoire d’Archéologie Moléculaire et Structurale (LAMS), CNRS, Sorbonne Université, 75005 Paris, France; laurence.de_viguerie@upmc.fr; 2 Laboratoire de Chimie Physique-Matière et Rayonnement (LCPMR), UMR 7614, CNRS, Sorbonne Université, 75005 Paris, France; tsveta.miteva@sorbonne-universite.fr; 3 Microsoft, Bellevue, WA 98004, USA; n.rohani@gmail.com

**Keywords:** reflectance imaging spectroscopy, hyperspectral imaging in the short-wave infrared range, deep neural network, pigment mapping, thangkas

## Abstract

Hyperspectral reflectance imaging in the short-wave infrared range (SWIR, “extended NIR”, ca. 1000 to 2500 nm) has proven to provide enhanced characterization of paint materials. However, the interpretation of the results remains challenging due to the intrinsic complexity of the SWIR spectra, presenting both broad and narrow absorption features with possible overlaps. To cope with the high dimensionality and spectral complexity of such datasets acquired in the SWIR domain, one data treatment approach is tested, inspired by innovative development in the cultural heritage field: the use of a pigment spectral database (extracted from model and historical samples) combined with a deep neural network (DNN). This approach allows for multi-label pigment classification within each pixel of the data cube. Conventional Spectral Angle Mapping and DNN results obtained on both pigment reference samples and a Buddhist painting (thangka) are discussed.

## 1. Introduction

Works of art are created using a wide variety of materials, or mixtures of materials, and often exhibit heterogeneities at multiple length scales. Historical paintings in particular consist of a superposition of paint layers, each prepared by grinding and mixing specific hybrid formulations (with various mineral pigments and organic binders) [1]. Their study thus requires the use of complementary analytical imaging techniques to probe and image the intrinsic complexity of such objects. Given the value and the uniqueness of cultural heritage artifacts, researchers favor analytical methodologies that can be used non-invasively and in situ (within cultural institutions, archaeological fields, etc.) [2]. As such, the last few years saw a tremendous rise in hyperspectral reflectance imaging spectroscopy in the cultural heritage domain [3,4]. This success may be attributed to the merits of the technique: it is non-invasive, portable, and allows for the wide field imaging of an artwork in under a few minutes. Hyperspectral imaging in the visible range (400–900 nm) is now a well-established technique to map the distribution of colorants across a painted surface. In the short-wave infrared range (SWIR, “extended NIR”, ca. 1000 to 2500 nm, i.e., 10,000 to 4000 cm^−1^), increased applications are being published in the field, as the SWIR region is of peculiar interest for the identification of pigments [5,6,7,8] but also organic binders and resins [9,10,11], with harmonics and combinations of the absorption bands observed in the mid-infrared range as well as absorption bands due to electronic transitions [12,13]. Most notably, pigments strongly absorbing in the near-infrared range present characteristic OH combination bands (i.e., hydrate minerals) or low-energy electronic transitions (i.e., Co- and Fe-based compounds), which allows for additional and complementary information on the pigment palette used by an artist. The main challenge remains to extract information from the large datasets created (millions of recorded spectra) in this energy domain, where the observed spectral outline typically emerges from multiple overlapping contributions especially in the case of complex mixtures and overlays of paint materials. While innovative data reduction and classification approaches in the visible range are growing to determine and map significant endmembers within hyperspectral dataset, a few works are dedicated to similar applications in the SWIR domain. Principal component analysis (PCA) and fully constrained least-squares (FCLS) spectral unmixing algorithms recently extended the use of multivariate analysis for automatic pigment and binder classification in this energy range [14,15,16]. However, the mapping of determined pigments/binders of interest still results in a challenging task as it necessitates performing nonlinear unmixing on large and complex datasets [17,18]. In this context, deep neural networks (DNNs) have recently proven to provide new opportunities in determining the nonlinear mappings between an input and the corresponding output automatically [19,20]. Remote sensing and hyperspectral imaging have more particularly benefitted from many DNN applications such as feature extraction, classification, and unmixing. The recent use of a DNN in cultural heritage for pigment identification in the visible range has paved the way to solve similar problems with an unprecedented processing time and accuracy rate [21,22]. In this paper, we thus propose to explore the use of DNN models for automatic pigment identification in SWIR hyperspectral datasets.

In order to improve the robustness of the model, an extended training dataset was built. Whereas in previous papers the dataset was constructed from regions of well-characterized paintings [21] or from pigment libraries [20], here we explore the combined use of various input data, selected from pure pigment pellets, paint mockups, and historical samples. It allows for the training library to incorporate the inherent variability of the dataset, a crucial step to improve the DNN prediction.

Paintings from one mockup and one historical thangka are used to test the robustness of the model. Thangkas are sacred paintings depicting a Buddhist deity; their main constituent materials are quite well known and described in the literature (thanks to literary sources [23,24], oral testimonies of modern-day painters but also previous analytical results [25,26]) as cotton cloth or silk, natural glue sizing and a coating traditionally composed of chalk or kaolinite, and several colored layer(s) made of mineral pigments and organic dyes.

The obtained DNN results are assessed in two ways. First, the accuracy of the model is calculated on a test dataset to evaluate the performance of the model. Second, the results are compared to those obtained via spectral classification followed by labeling of the pigments present based on complementary information from visible hyperspectral imaging (VIS-RIS) and single-point X-ray fluorescence (XRF).

## 2. Materials and Methods

### 2.1. Short-Wave Infrared Hyperspectral Imaging (SWIR)

SWIR hyperspectral imaging was carried out by a camera (Specim Corp, Oulu, Finland) SWIR 3 equipped with an OLES 30 lens (focal length: 56 mm) and mounted on a motorized translation to ensure the analysis of the painted surface with a working distance of 1.0 m. The camera is a push-broom imaging device coupled with a cryogenically cooled MCT detector (384 (spatial) × 288 (spectral) pixels), operating in the 1000–2500 nm range with a 12 nm spectral resolution, and a 5.6 nm spectral sampling. Diffuse illumination was provided by two 20 W halogen lamps, half a meter away from the painting. The dataset was calibrated to apparent reflectance by subtracting a dark image from the collected reflectance data in digital counts, and dividing it by the illumination irradiance (acquired on a 99% reflectance white reference, Spectralon, LabSphere Inc., North Sutton, NH, USA). The reflectance spectra were used as input data to the DNN model.

### 2.2. Visible Near-Infrared Hyperspectral Imaging (VIS-RIS)

VIS-RIS hyperspectral camera (Specim Corp, Oulu, Finland) mounted on a motorized translation, was used with a spectral sampling of 2.8 nm over the 400–1000 nm spectral range. Illumination was provided by two 20 W halogen lamps 1 m away. Spectral Angle Mapper results were obtained using the dedicated ENVI software (Harris Corporation, Melbourne, FL, USA).

### 2.3. Single Point X-ray Fluorescence (XRF)

XRF was performed using an in-house instrument, equipped with a Pd anode transmission tube (Moxtek MAGNUM, Orem, Utah) operated at 30 kV and 0.1 mA (3 W) and a Silicon Drift Detector (active area of 25 mm^2^, X-123FAST SDD, Amptek, Bedford, MA, USA). The beam size was approximately 1.2 mm for a typical working distance of 1 cm. The system operated with acquisition time of 300 s. For the evaluation of the raw full spectral XRF data, the dedicated PyMCA software [27] was used.

### 2.4. Samples

A set of eight pigments (Kremer Pigmente, Aichstetten, Germany), traditionally used in thangkas, were prepared as single pigment pellets: malachite (10300) Cu_2_CO_3_(OH)_2_, azurite Cu_2_(CO_3_)_2_(OH)_2_ (10200), vermilion HgS (42000), orpiment As_2_S_3_ (10700), minium Pb_3_O_4_ (42500), indigo (36000), red aluminum lake of carminic acid (42100—extracted from cochineal), calcite CaCO_3_ (58720), and kaolinite Al_2_Si_2_O_5_(OH)_4_ (58200). X-ray diffraction was preliminarily carried out to confirm the composition of crystalline compounds.

**Figure 1:**
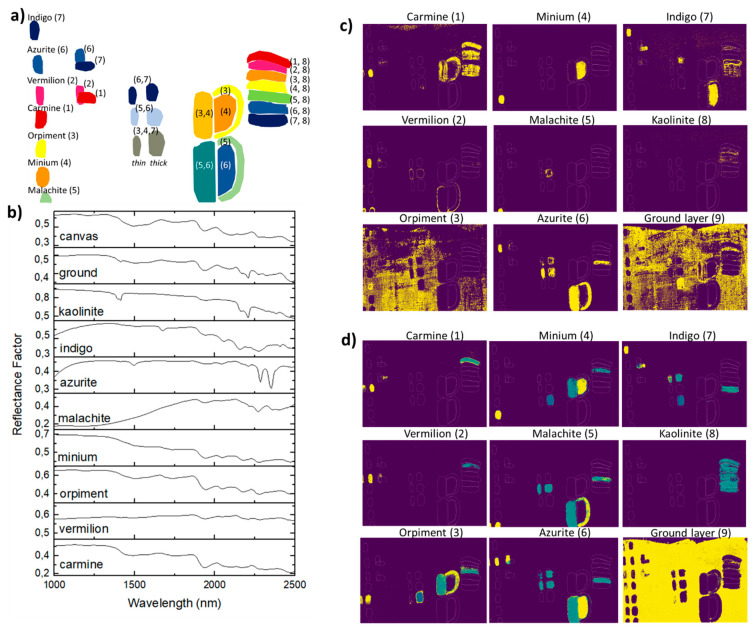
(**a**) Schematic view of a set of mockup paintings composed of: 8 single-pigment paint systems and a ground layer (namely carmine (1), vermilion (2), orpiment (3), minium (4), malachite (5), azurite (6), indigo (7), kaolinite (8), and ground layer (9)), and 14 mixtures using two pigment types (the mixtures can be split into four groups: (i) 7 mixtures of two colors composed of kaolinite white pigment and a non-white pigment with a 1:2 wt%, (ii) 4 mixtures of two non-white pigments with a 1:2 wt%, (iii) 1 mixture of three non-white pigments with a 1:3 wt%, and (iv) 2 overlays of two single pigment layers); (**b**) average reflectance spectra of all single-pigment paint layers applied on the prepared canvas; (**c**) pigment classification maps obtained using SAM; (**d**) pigment coefficient maps obtained using DNN model.

A model thangka was prepared using the same set of reference pigments (Figure 1a and Figure S1). For the support, Indian cotton cloth was prepared using the following procedure: Shika Nikawa glue was applied first (Uematsu, fine art material shop, Tokyo, Japan), once dry (24 h) the surface was sanded, then a mixture of glue with kaolinite, chalk, and earth pigments was applied [24]. The Kremer reference pigments were then weighted and mixed with two drops of glue before application on the prepared canvas. The layer was thickly applied to obtain visible coverage of the support; however, no measurement of the layer thicknesses was performed. In total, the system presents 8 single-pigment paint systems and a ground layer (namely carmine (1), vermilion (2), orpiment (3), minium (4), malachite (5), azurite (6), indigo (7), kaolinite (8), and ground layer (9)), and 14 mixtures using two pigment types (the mixtures can be split in four groups: (i) 7 mixtures of two colors composed of kaolinite white pigment and a non-white pigment with a 1:2 wt%, (ii) 4 mixtures of two non-white pigments with a 1:2 wt%, (iii) 1 mixture of three non-white pigments with a 1:3 wt%, and (iv) 2 overlays of two single pigment layers).

**Figure 2:**
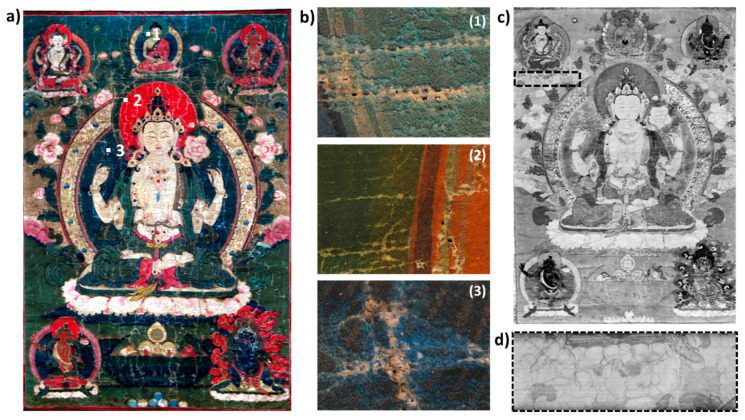
(**a**) RGB image reconstructed from VIS-RIS dataset (red channel is assigned to 639.1 nm, green to 550.6 nm, and blue to 460.6 nm) of the historical tangka analyzed (size: 70 × 120 cm^2^). Three blue and green areas are located that refer to: (**b**) macro-photographs of three locations highlighting intrinsic mixture of blue and green particles, and surface degradation; (**c**) short-wave infrared imaging data at 1650 nm that reveals (**d**) the underlying preparatory drawing and contour of a floral shape, located using dashed rectangle in (**c**).

One historical Tibetan thangka (Figure 2a, size: 70 × 120 cm^2^) dated from the beginning of the 19 th century was analyzed, which belongs to a French private collection. It represents Chenrezig, the Tibetan incarnation of Avalokitesvara, the bodhisattva of compassion.

### 2.5. DNN Model

#### 2.5.1. Overall DNN Workflow

A classical workflow to create the model using a neural network trained and tested on an appropriate dataset is established. The pipeline of the approach to produce labeled qualitative pigment maps follows three main steps. First, a large spectral training dataset is prepared, and each input spectrum is labeled with the related pigment class(es) and relative mass ratio(s). Second, the neural network is trained on 80% of the original dataset to predict the pigments present in the input RIS spectra. The remaining 20% of the original dataset is split into two—a validation and a test set. The validation set is used to determine the optimal neural network architecture and for hyperparameter tuning. The test set is used to estimate the overall classification accuracy of the neural network (predictions of pigments present).

Lastly, the neural network prediction of pigments is evaluated on two case painting studies: the entire model system and one historical thangka. The output of the model consists of 11 labels and coefficient maps, one for each of the classes in the training dataset, i.e., azurite, malachite, minium, kaolinite, vermilion, orpiment, carmine, indigo, calcite, Indian cotton support, and unidentified white paint historical layer. For the label maps, each pixel value of the composite pigment class map is set to 0 or 1, which corresponds to the absence or presence of a class in a determined pixel, respectively. The presence of a pigment in a given pixel is obtained using a threshold of 0.5 or greater over the probability value determined by the DNN model of a match between the SWIR spectrum and the pigment class. The threshold is chosen to reduce the number of false-positive identifications while providing a higher accuracy value. The output coefficient maps provide a qualitative mapping of the abundance of the pigment classes.

#### 2.5.2. DNN Input Dataset

In this paper, we combine three different types of input data to train and test the DNN model. Data are extracted from (i) single-pigment pellets, (ii) single pigment, 2- and 3-pigment mixture paint layers, (iii) the canvas and prepared canvas of the mockup sample, and finally (iv) specific areas of the historical tangka preliminarily characterized by single-point XRF and VIS-RIS. In total, the input dataset represents 12,000 spectra with a number of evenly distributed samples per class.

#### 2.5.3. DNN Architecture

Pigment identification is modeled as a multi-label classification problem, where each reflectance spectrum can have multiple labels due to the presence of multiple pigments. For semi-quantitative mapping, the spectral unmixing task is solved in parallel to the multi-label classification, using a different input—namely the respective K/S values extracted from the reflectance spectra using the Kubelka–Munk theory [28].

The deep learning model used in this work was taken from [20]. It consists of two identical deep feed-forward networks, which are simultaneously trained. The two DNNs consist of four fully connected hidden layers with 256, 128, 64, and 32 hidden nodes and an output layer with 11 nodes with ReLU/Sigmoid activation functions (see Figure S2).

In the case of the pigment identification DNN, the units in the output layer correspond to different pigment classes. In the case of pigment unmixing, a second output layer of 11 units is added. It is obtained by multiplying the output layers of the two parallel DNNs for pigment identification and unmixing, and applying a softmax activation function. This output layer corresponds to the estimated coefficient vector α of pigment weight concentrations for the given spectrum. To train the networks proposed for pigment classification and pigment unmixing, we use the binary cross-entropy and the Kullback–Leibler Divergence (KLD) [29] loss functions, respectively. The Adam optimizer with default configuration parameter values, e.g., a learning rate of 0.001, is used to optimize the objective loss functions.

Python utilizing the Keras library [30] with Tensorflow [31] as the backend is used to define and train the model. The number of epochs and the batch size are set to 200 and 64, respectively. To avoid overfitting, an Early Stopping callback is used and the number of patience is set to 10.

The training of the DNN model takes 71 s on a single Intel(R)-Core(TM) it-5600U (CPU @ 2.60 GHz). Subsequently, obtaining the pigment map of the historical tangka takes about 20 s on the same hardware architecture.

### 2.6. Spectral Angle Mapping Algorithm (SAM)

SAM measures the spectral similarity between spectra. The algorithm determines the angle between two spectra, a reference (or endmember) and an experimental spectrum considered as vectors in a space with n-dimensions equal to the number of spectral sampling. For a 3D data cube, SAM compares a matrix of reference spectrum vectors to each pixel vector in the spectral sampling space. As such, for each pixel, a smaller angle value represents a closer match to a reference spectrum [32].

The spectra used as references in this study are defined as the average per class of the NN input mockup pure pigment layers (Figure 1b). The spectral angles are computed for each class within all pixels using Spectral Python (SPy) [33]. The class presenting the smallest spectral angle between its reference spectrum and the pixel spectrum is set to 1, and the remaining classes are set to 0. Thus, the output of SAM consists of 11 binary label maps.

## 3. Results

### 3.1. Comparison of DNN versus SAM for Pigment Classification and Mapping Tasks

The efficiency of the SAM and DNN approaches to classify the different pixels of the mockup sample dataset are compared in Figure 1c and Figure 1d, respectively.

The efficiency of the SAM approach for data classification is first determined on a selection of labeled pure pigment layers (pure thick layer on the left side of the model sample, Figure 1b) from which the SAM reference spectra were extracted. The approach provides classification with an accuracy of 93%. SAM is then applied for the classification of the entire mockup dataset composed of pure pigments and mixtures. Several limitations of the approach are observed (Figure 1c). The pure orpiment layers and large portions of the prepared canvas are classified together, and labeled as orpiment. Whereas the add-in of kaolinite pigment results in absorptions at ca. 1390, 1410, 1910, and 2200 nm [26], most pigment mixtures with kaolinite are mislabeled, and are assigned to the carmine class. Similarly, for two- and three-pigment mixtures, azurite is the only pigment properly labeled; moreover, in the specific case of azurite and malachite mixtures, pixels are systematically labeled as azurite, malachite being rarely identified in the mixture. The SAM classification also fails at classifying pure pigment layers from which the reference spectra were not extracted (layers on the right side of the mockup), as such, the pure layer of azurite is labeled as indigo, and the pure layer of malachite is labeled as azurite.

Based on these results, SAM provides good accuracy on the area where the endmembers were defined. However, the pigments present in mixtures and other single-pigment layer areas are not properly classified. Thus, the approach requires good a priori knowledge of the expected endmember prior to the classification, and class mapping should present very restrictive threshold values to prevent false-positive classification results.

When the DNN model is used to process the full dataset, more representative and accurate classification is obtained (Figure 1d and Figure S3). The classification accuracy of the DNN model is estimated to be 98.1% for the testing dataset containing pure pigments and pigment mixtures. The results provide efficient mapping of pigments of interest, e.g., azurite and malachite, but also semi-conductor pigments such as minium and orpiment. Tints of colored pigments mixed with kaolinite, pigment mixtures, and paint layer overlays are labeled accordingly to their expected classes. Thus, the DNN classification provides a straightforward and qualitative mapping of the pigment and pigment mixture signatures present within the data cube.

In the region from 1000 to 2500 nm, the approach was expected to provide efficient mapping of pigments that present overtones and combination bands associated with vibrational transitions of molecular bonds involving mainly hydrogen, carbon, nitrogen, oxygen, and related functional groups. As an example, with characteristic absorption maximum in the NIR region, corresponding to a [ν_2_ (OH)] overtone at 1497 nm, a superposition band of stretching and bending modes [(ν + δ) OH] at 2289 nm and a stretching overtone at [ν_2_ (OH)] at 2352 nm, azurite is accurately mapped for both pure and mixed systems. However, for semi-conductor type pigments ruled by electronic transitions with selective absorption in the visible region, the DNN approach also provided efficient labeling and mapping of both pure pigments and pigment mixtures. As such, the reflectance spectra of the chemical compound present characteristic spectral variation apprehended by the model. As an example, the appropriate classification of the pixels containing minium could be explained by a variation of its absorption coefficient in the SWIR domain. For spectra acquired on pellet and mockup samples, a broad decrease in reflectance intensity was observed from 1330 to 1800 nm. Further work should be specifically carried out to determine the origin of such intensity variations and identify the spectral behavior of semi-conductor pigments in mixtures and superimpositions. 

### 3.2. The Use of Deep NN for Classification Task in Historical Tangkas

Following the results on mockup samples, the mapping of several pigment classes is obtained using the DNN model on a historical Buddhist painting (Figure 2a), previously studied by single-point XRF and VIS-RIS. Here, we combine the results of the multiple spectroscopic techniques for a deeper understanding of the materiality of historical thangkas.

**Figure 3:**
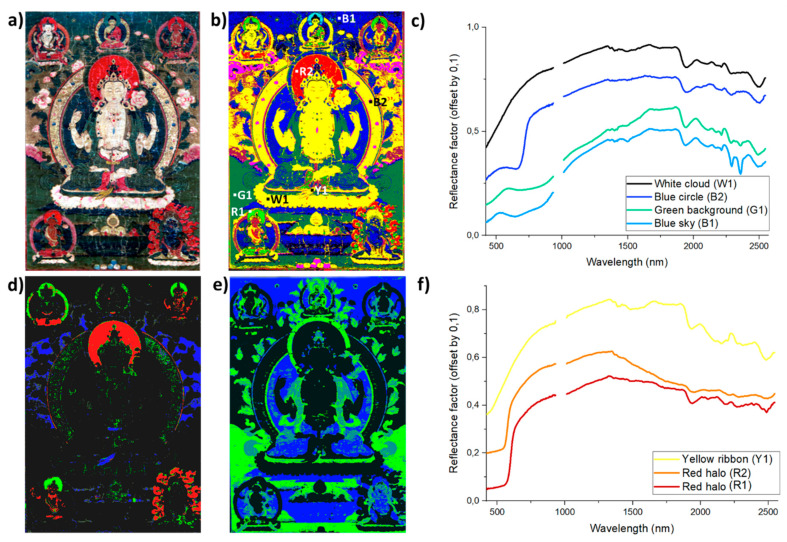
(**a**) Visible picture of the historical tangka analyzed; (**b**) SAM classification of VIS-RIS dataset (red: minium, blue: azurite, light green: vermilion, dark green: malachite, pink: indigo, brown: organic lake—tolerance angle 0.15 rad) with analyzed sites and corresponding reflectance spectra presented in (**c**,**f**); DNN model results for (**d**) minium, vermilion, orpiment (in red, green and blue, respectively) and (**e**) azurite and malachite (in blue and green, respectively).

The blue and green areas of the painting present visible signs of degradation at the surface of the object. As already discussed in [26], a strong darkening of azurite is very commonly observed on thangkas, although not chemically characterized yet. However, the observation of lacunas in degraded areas reveals the presence of undegraded paint material underneath the top layer (a few microns thick) with an intricate mixture of blue and green particles (Figure 2b). As a consequence of the degradation state of the thangka, the obtained VIS-RIS spectra do not allow the accurate identification of the original pigments, though Cu is identified in large amounts by p-XRF. The SWIR data show the presence of overtones and combinations of stretching and bending modes of O–H groups and carbonate ions, characteristics of the Cu-based pigments used in the paint layers (no characteristic absorption of Cu-based degradation products was identified in the data analyzed) [8,17,34]. More specifically, the deep blue color of the sky as well as the body and aura of some divinities are painted using the azurite (Cu_2_(CO_3_)_2_(OH)_2_) pigment, characterized by the presence of absorption bands at 1491, 2285, and 2352 nm (Figure 3c). The green pigment mainly used to paint the background, the leaves, and auras of divinities is identified as malachite (Cu_2_CO_3_(OH)_2_), with characteristic absorption features at 2274 and 2352 nm (Figure 3c). The results of the DNN model allow for the identification of both compounds, and provide more subtle information about their spatial distribution and mixtures (Figure 3a,b,e). First, azurite is mainly found pure in the blue areas. Second, the decorative leaves surrounding the top central divinity, not identified by VIS-RIS, are correctly mapped and labeled as malachite-rich paint layers. Third, whereas the green background and the central aureole of the main deity present a higher content of malachite (confirmed by the lowest absorption in the 2320–2380 nm range), green leaves as well as the main deity’s clothes present various contents of both malachite and azurite pigments, which confirms their combined use in several areas of the painting.

For the red areas, two different pigments are identified thanks to p-XRF and VIS-RIS data, namely minium (XRF: Pb—VIS inflection point: 564 nm) and vermilion (XRF: Hg, S—VIS inflection point: 590 nm). The DNN-based mapping is consistent with the location proposed in the visible range (Figure 3b,d). Minium (Pb_3_O_4_) is properly identified as the main pigment in the aureole of the central divinity, some clothing parts of the smaller divinities, and in the red decoration surrounding the bottom-right figure. As described previously, a decrease in the reflectance spectrum is observed from 1330 to 1800 nm, and this feature is redundant and characteristic of red areas painted using minium pigment (Figure 3f, spectrum R2). Vermilion presents a constant absorption coefficient over the SWIR domain. The absence of absorption features, with constant reflection intensity, results in a good mapping of the vermilion-rich paint layers in the halos of two divinities as well as in specific parts of the decoration surrounding the bottom-right divinity (Figure 3f, spectrum R1).

For the yellow area, the presence of As confirmed by p-XRF and the presence of an inflection point located at 500 nm in the VIS-RIS spectra indicate the use of an orpiment pigment. The DNN model allows the mapping of the As-based compound, in accordance with VIS-RIS and p-XRF results (Figure 3b,c), in the ribbons at the level of the feet and belly of the central deity, as well as in the light brown background. The model properly predicts all orpiment-based areas. In the case of the orpiment, a relatively high reflectance factor in the 1500–1900 nm range compared to the 2000–2400 nm energy domain is observed for both the mockup material and historical dataset, and could explain the appropriate labeling of the pigments (Figure 3f, spectrum Y1). Here, again, more work on the absorption coefficient of the pigment in mixtures and layered systems should be performed.

The SWIR signals in the white and pink areas are similar and present absorption bands at 2275, 2105, and 1930 nm, and a very broad band centered at ca. 1490 nm (Figure 3c, spectrum W1). Their positions are characteristic of cellulose fibers (such as cotton, most probable in the case of Tibetan artworks) [26]. Vibrational features from the (C = O), N–H, and CH_2_ groups of the animal skin glue, which include features near 2041 nm and 2176 nm, can be differentiated [11]. Other characteristic peaks at 1720 and 2170 nm are observed in this spectrum, which could be attributed to the presence of gypsum; however, the absorption bands are relatively weak. It was not possible to more precisely identify the white pigment, whose spectrum probably results from the overlay of a cellulose-based material, with a layer of kaolinite-type clay and gypsum mixed with animal glue.

Interestingly, two areas below the top corner figures, clouds with parts painted in blue and identified as a thin layer of indigo in the visible range (with an apparent absorbance maximum at 660 nm), are labeled as carmine-rich paint material (Figure S4). The two areas overlap with an underlying floral composition (visible in SWIR at 1650 nm—Figure 2c,d). This can suggest the use of a red dye-based paint, being the original color of this area. This will initiate new research to more specifically look for underlying composition in similar works of art.

## 4. Conclusions

The DNN approach proposed in this study offers new possibilities in the SWIR range to identify and map pigments in complex materials either for unknown mixtures or multi-layered systems. Applied to both mockup and historical paintings, the DNN model has been proven to outperform the most common classification technique in the field known as SAM. DNN allows a more representative mapping of the pigments of interest, which results from the multi-labeling proposed by the approach (i.e., a single pixel can contain several classes) and the multiple inputs from mockup and historical paintings used as a training dataset.

This study confirms the potential of SWIR for pigments with characteristic absorption features such as malachite and azurite, even when they are mixed and in degraded systems. In the case of semi-conductor-type pigments, more research is needed to understand the overall spectral shape/intensity in the SWIR domain, but it is definitely to be considered as it allows efficient mapping of such pigments and brings complementary information to the VIS-RIS spectra, in particular when multi-layered systems are investigated.

As recently outlined in numerous studies, combined access to VIS and SWIR imaging modalities improves pigment classification in historical datasets; it is thus expected that neural network models trained on combined datasets will solve more challenging tasks in the near future.

## Data Availability

Data available on request due to restrictions eg privacy or ethical. The data presented in this study are available on request from the corresponding author.

## Supplementary Materials

The following are available online at https://www.mdpi.com/article/10.3390/s21186150/s1, Figure S1: Comparison of (a) the visible picture, and (b) schematic view of the laboratory-prepared thangka composed of: 8 single-pigment paint systems and a ground layer (namely carmine (1), vermilion (2), orpiment (3), minium (4), malachite (5), azurite (6), indigo (7), kaolinite (8), and ground layer (9)), and 14 mixtures using two pigment types (the mixtures can be split into four groups: (i) 7 mixtures of two colors composed of kaolinite white pigment and a non-white pigment with a 1:2 wt%, (ii) 4 mixtures of two non-white pigments with a 1:2 wt%, (iii) 1 mixture of three non-white pigments with a 1:3 wt%, and (iv) the remaining 2 mixtures are overlays of single pigment layers), Figure S2: Deep neural network architecture of the model used for simultaneously solving the pigment identification and unmixing tasks (see DNN model part for more details), Figure S3: Confusion matrix of the pure pigments in the test set, Figure S4: Confusion matrix of the pigment mixtures in the test set. Figure S5: Pigment coefficient maps obtained using DNN model for the historical thangka SWIR dataset, for azurite, malachite, minium, kaolinite, vermilion, orpiment, carmine, and the white paint layer. Table S1: Precision and recall values for the pure pigments in the test set, Table S2: Precision and recall values for the pigment mixtures in the test set.
