# Supplementary material for: Artificial Intelligence for Pigment Classification Task in the Short-Wave Infrared Range
Source: Sensors (Basel). 2021 Sep 13;21(18):6150. doi: 10.3390/s21186150 (PMC8471921; doi:10.3390/s21186150)
Supplement: Supplementary file 1 [file sensors-21-06150-s001.zip › sensors-1322816-supplementary.pdf]

Supplementary Information:

Figure S1 Comparison of a) the visible picture, and b) schematic view of the laboratory prepared thangka composed of: 8 single-pigment paint systems and a ground layer (namely carmine (1), vermilion (2), orpiment (3), minium (4), malachite (5), azurite (6), indigo (7), kaolinite (8), and ground layer (9)), and 14 mixtures using two pigment types (the mixtures can be split in four groups: (i) 7 mixtures of two colors composed of kaolinite white pigment and a non-white pigment with a 1:2 wt %, (ii) 4 mixtures of two non-white pigments with a 1:2 wt % , (iii) 1 mixture of three non-white pigments with a 1:3 wt %, (iv) the remaining 2 mixtures are overlays of single pigment layers).

a)

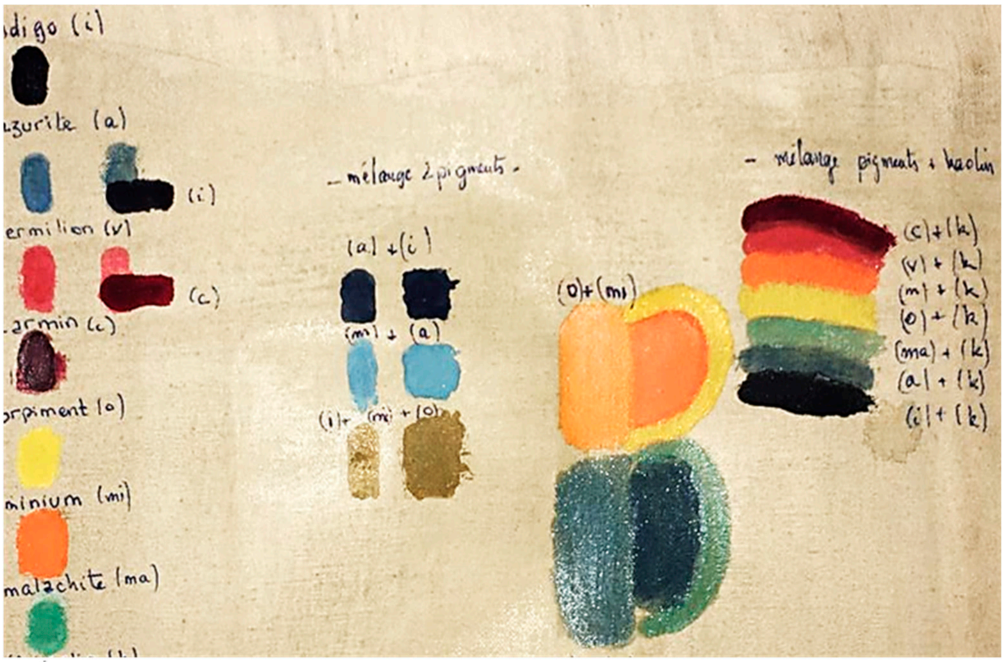

b)

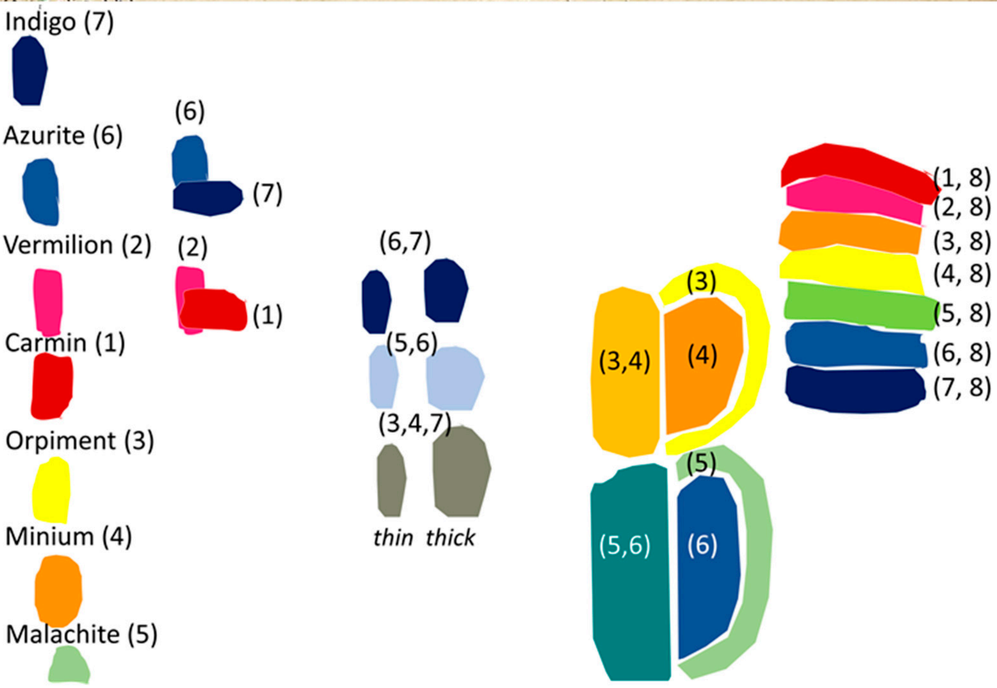

Figure S2 Deep Neural Network architecture of the model used for simultaneously solving the pigment identification and unmixing tasks (see DNN model part for more details).

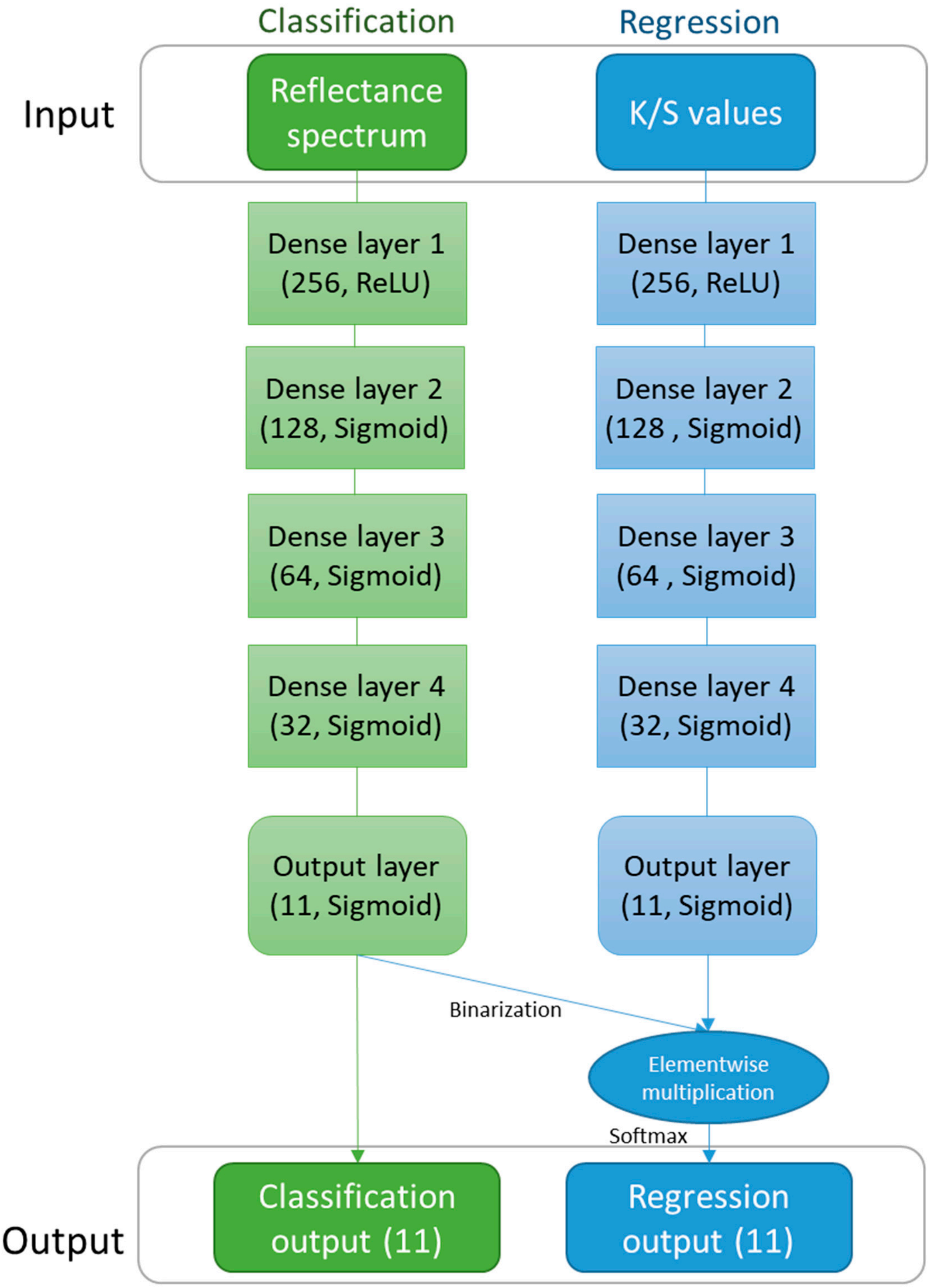

Pigment mapping DNN results

The total accuracy of pigment prediction on the test set is 97% (515 out of 530 samples predicted correctly). The model has 98% accuracy in the prediction of pure pigments (393 out of 401), and 95% accuracy in the prediction of mixtures of pigments (122 out of 129). The confusion matrices for the pure pigments and mixtures of pigments of the test set are presented below—they contain all correctly predicted pigments and mixtures as well as the misclassified examples.

Figure S3 and Table S1 show the confusion matrix as well as the values of the precision and recall for the pure pigment paint systems. Out of the eight misclassified samples, there are six cases where the model predicts two pigments instead of one (in the cases of carmine and malachite, a mixture of these two pigments with kaolinite, and in the case of orpiment, a mixture of orpiment and minium), and two cases where it predicts a single pigment (minium and calcite instead of orpiment).

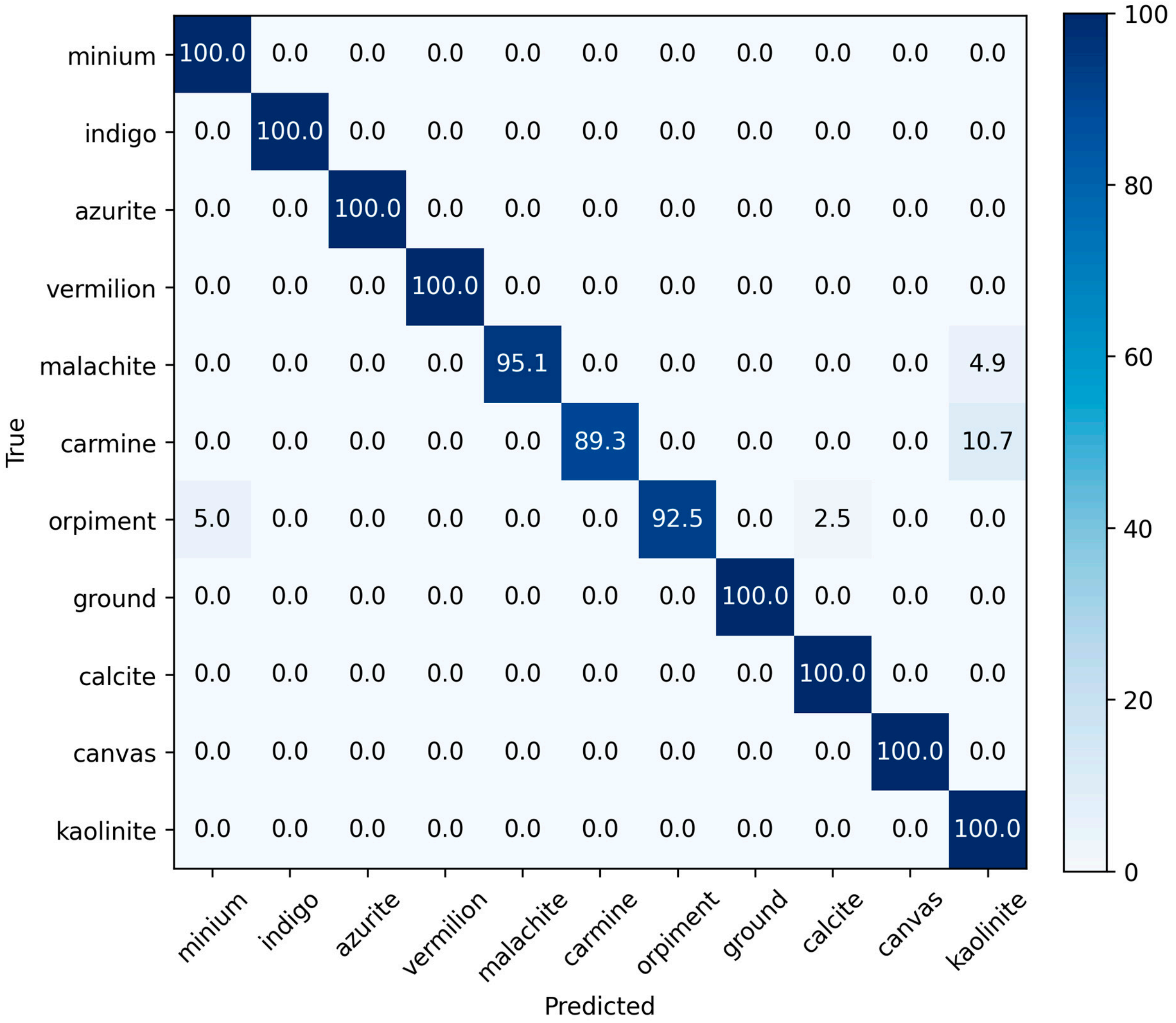

Figure S3 Confusion matrix of the pure pigments in the test set.

Table S1. Precision and recall values for the pure pigments in the test set.

	Precision	Recall
Minium	0.97	1.00
Indigo	1.00	1.00
Azurite	1.00	1.00
Vermillion	1.00	1.00
Malachite	1.00	0.95
Carmine	1.00	0.89
Orpiment	1.00	0.93
Ground	1.00	1.00
Calcite	0.95	1.00
Canvas	1.00	1.00
Kaolinite	0.85	1.00

In the case of pigment mixtures, the misclassified test samples are a result of the model predicting one instead of two pigments. This is shown in the confusion matrix (Figure S4) and the table containing the precision and recall values (Table S2). The misclassified pigment mixtures are carmine + ground layer, vermilion + kaolinite, and minium + orpiment. They are wrongly classified as ground layer only (carmine + ground, vermilion + kaolinite) and minium only (minium + orpiment).

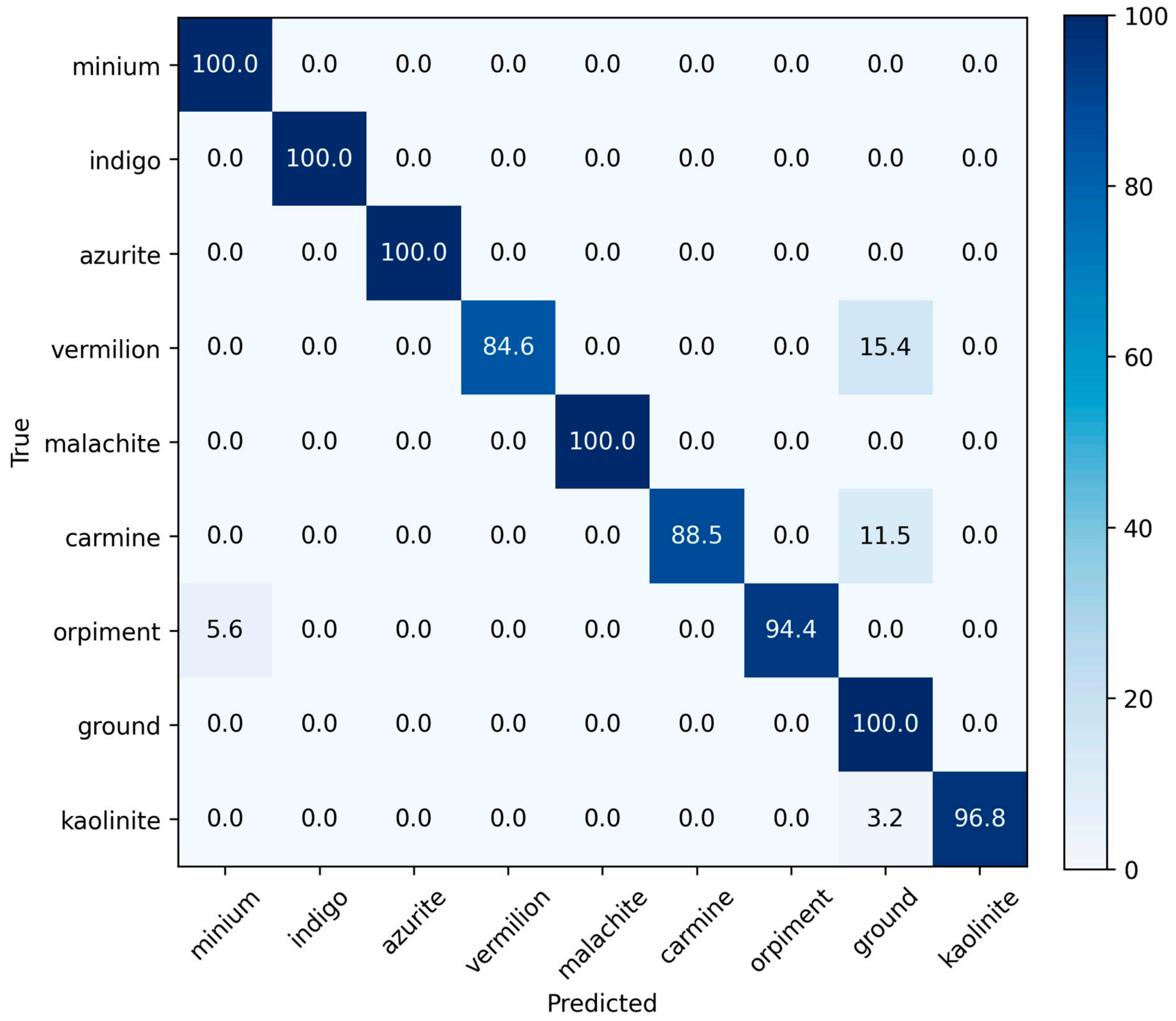

Figure S4 Confusion matrix of the pigment mixtures in the test set.

Table S2 Precision and recall values for the pigment mixtures in the test set.

	Precision	Recall
Minium	0.95	1.00
Indigo	1.00	1.00
Azurite	1.00	1.00
Vermillion	1.00	0.85
Malachite	1.00	1.00
Carmine	1.00	0.88
Orpiment	1.00	0.94
Ground	0.68	1.00
Calcite	1.00	0.97
Canvas	0.95	1.00
Kaolinite	1.00	1.00

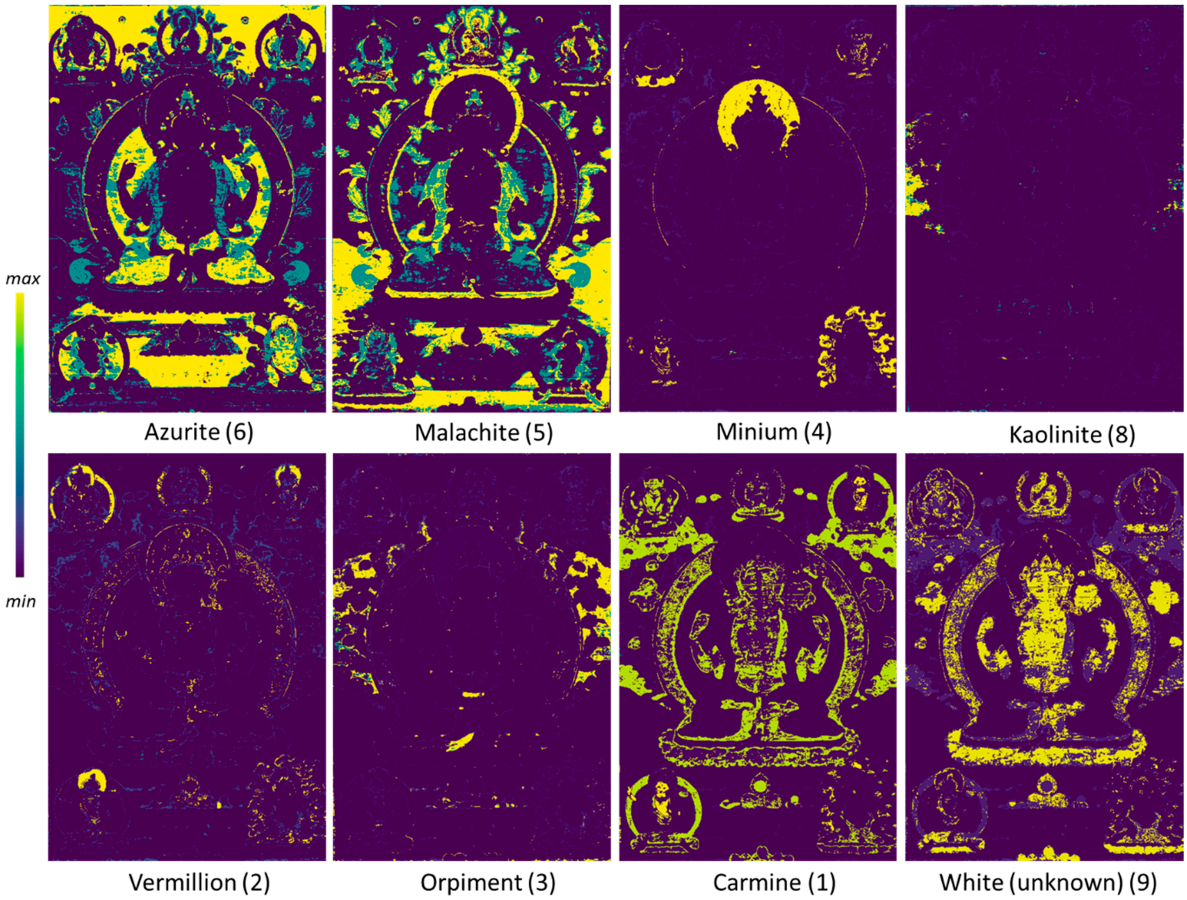

Figure S5 Pigment coefficient maps obtained using DNN model for the historical thangka SWIR dataset, for azurite, malachite, minium, kaolinite, vermilion, orpiment, carmine, and the white paint layer.
